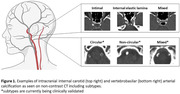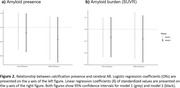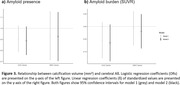# Determining the association between intracranial arterial calcification and cerebral amyloid deposition – A population imaging‐based approach

**DOI:** 10.1002/alz.088736

**Published:** 2025-01-09

**Authors:** Anna M Streiber, Julia Neitzel, Phuong Thuy Nguyen Ho, Meike W. Vernooij, Daniel Bos

**Affiliations:** ^1^ Erasmus University Medical Center, Rotterdam Netherlands; ^2^ Harvard T.H. Chan School of Public Health, Boston, MA USA

## Abstract

**Background:**

Arteriosclerosis is linked to an increased risk of dementia and Alzheimer’s disease. However, the underlying pathological mechanisms remain unclear. We investigated the association between intracranial arteriosclerosis and cerebral Amyloid beta (Aß). Calcification of the intracranial internal carotid‐ (ICAC) and the vertebrobasilar arteries (VBAC) were used as a proxy of arteriosclerosis. We hypothesized that calcification presence, burden, and subtypes, differentially relate to changes in cerebral Aß.

**Method:**

Analyses were based on 633 participants from the population‐based Rotterdam Study (mean age 69.3 ± 5.46 years, 51.2% female) who underwent an amyloid PET‐CT scan to quantify both calcification in the intracranial arteries (on low dose CT) and cerebral Aß accumulation. Calcification volume (in mm^3^) and subtypes (Figure 1) have been assessed by two independent raters. Aß burden was quantified using the standardized uptake value ratio (SUVR). Based on a clinically defined SUVR cutoff, participants were classified as Aß positive or Aß negative. The association between the presence, burden, and type of intracranial calcification and Aß was assessed by applying logistic and linear regression models. In Model 1, we adjusted for age, sex and APOE‐e4 carriership. In Model 2, we additionally corrected for cardiovascular risk factors.

**Result:**

In total, 78.4% of the participants presented with ICAC and 13.3% with VBAC. In 16.43% of the participants, we detected Aß levels that were above the defined SUVR cutoff. Overall, calcification presence, and higher calcification volume were associated with lower odds of being Aß positive and a lower Aß SUVR (Figure 2 and 3). However, none of the results were statistically significant. The results did not differ per calcification subtype. We employed sensitivity analyses to account for potential selection bias and confounding, which did not change the overall results.

**Conclusion:**

We found no significant association between intracranial arterial calcification and cerebral Aß accumulation. Future research should explore other potential neuropathological links to further understand the etiology underlying Alzheimer’s disease.